# Supplementary material for: Depression in Central and Eastern Europe: How Much It Costs? Cost of Depression in Romania
Source: Healthcare (Basel). 2023 Mar 22;11(6):921. doi: 10.3390/healthcare11060921 (PMC10048715; doi:10.3390/healthcare11060921)
Supplement: Supplementary file 1 [file healthcare-11-00921-s001.zip › healthcare-2229053-supplementary.pdf]

Table S1. Components of depression costs

Year	2015	2016	2017	2018	2019	2020	2021
**Direct costs**							
Total number of cases N	277,317	306,290	343,171	378,447	404,935	394,860	435,772
Primary care (€)<br>(%)	17,954<br>(0.04)	14,144<br>(0.03)	12,289<br>(0.02)	15,274<br>(0.02)	16,887<br>(0.03)	32,717<br>(0.07)	22,155<br>(0.05)
Specialty outpatients (€)<br>(%)	3,394,070<br>(7.78)	4,700,617<br>(11.51)	5,682,662<br>(9.73)	6,431,881<br>(10.40)	7,184,106<br>(11.54)	7,774,020<br>(17.68)	8,785,719<br>(18.87)
Psychotherapy (€)<br>(%)	1,080,655<br>(2.48)	1,376,534<br>(3.37)	1,720,490<br>(2.94)	1,954,258<br>(3.16)	2,297,732<br>(3.69)	2,434,415<br>(5.54)	2,903,538<br>(6.24)
Hospitalization (€)<br>(%)	15,988,568<br>(36.67)	17,650,718<br>(43.24)	33,874,380<br>(57.97)	36,160,237<br>(58.48)	35,187,675<br>(56.51)	15,061,283<br>(34.26)	15,351,484<br>(32.97)
Medications (€)<br>(%)	23,120,678<br>(53.03)	17,081,027<br>(41.84)	17,139,546<br>(29.33)	17,268,898<br>(27.93)	17,578,008<br>(28.23)	18,661,982<br>(42.45)	19,505,236<br>(41.89)
Total of direct costs (€)	43,601,925	40,823,040	58,429,367	61,830,548	62,264,408	43,964,417	46,568,132
Average cost/patient/year (€)	158	133	170	164	155	112	108
**Indirect costs (€)**							
Number of sick leave	1,627,940	1,822,354	1,936,336	2,419,404	2,461,103	2,543,280	687,784
Sick leave (€)	301,512,782	363,438,423	431,847,125	629,324,610	763,943,807	920,210,070	251,938,419
Average cost/patient/year (€)	185	199	223	260	310	362	366
Sick leave cost/direct cost	**10.8**	**8.9**	**7.4**	**10.2**	**12.3**	**18.4**	**5.4**
**Number of deaths**							
Number of deaths<br>(%)	5032<br>(1.81)	10237<br>(3.34)	14857<br>(4.33)	19445<br>(5.14)	23443<br>(5.79)	31932<br>(8.09)	38394<br>(8.81)
Cost of productivity loss (€)	933,190,960	523,154,870	1,660,755,924	1,147,827,402	1,500,965,020	1,869,221,934	2,455,305,818

Table S2. Corelation matrix (Pearson (n))

Variables	Number of primary care	Number of specialty outpatients	Number of psychotherapy services	Number of hospitalization days	Number of sick leave	Number of Medical prescriptions	Cost of primary care (€)	Cost of specialty outpatients (€)	Cost of psychotherapy services (€)	Cost of hospitalizatin (€)	Cost of medications (€)	Cost of sick leave (€)	Cost of productivity loss (€)
Number of primary care	**1**	-0.104	-0.107	-0.437	-0.000	-0.258	**0.735**	-0.165	-0.138	**-0.799**	0.648	0.135	-0.021
Number of specialty outpatients	-0.104	**1**	**0.997**	-0.783	-0.132	**0.980**	0.573	**0.995**	**0.998**	-0.027	-0.264	0.360	**0.866**
Number of psychotherapy services	-0.107	**0.997**	**1**	**-0.783**	-0.184	**0.974**	0.559	**0.989**	**0.999**	-0.049	-0.229	0.312	**0.877**
Number of hospitalization days	-0.437	**-0.783**	**-0.783**	**1**	0.324	-0.661	**-0.833**	**-0.749**	**-0.762**	0.576	-0.080	-0.167	**-0.780**
Number of sick leave	-0.000	-0.132	-0.184	0.324	**1**	-0.035	0.086	-0.083	-0.152	0.507	-0.410	0.854	-0.342
Number of Medical prescriptions	-0.258	**0.980**	**0.974**	-0.661	-0.035	**1**	0.451	**0.989**	**0.983**	0.167	-0.372	0.409	**0.839**
Cost of primary care (€)	**0.735**	0.573	0.559	**-0.833**	0.086	0.451	**1**	0.530	0.540	-0.579	0.258	0.506	0.512
Cost of specialty outpatients (€)	-0.165	**0.995**	**0.989**	**-0.749**	-0.083	**0.989**	0.530	**1**	**0.994**	0.036	-0.353	0.389	**0.842**
Cost of psychotherapy services (€)	-0.138	**0.998**	**0.999**	**-0.762**	-0.152	**0.983**	0.540	**0.994**	**1**	-0.008	-0.265	0.336	**0.870**
Cost of hospitalization (€)	**-0.799**	-0.027	-0.049	0.576	0.507	0.167	-0.579	0.036	-0.008	**1**	-0.574	0.3006	-0.087
Cost of medications (€)	0.648	-0.264	-0.229	-0.080	-0.410	-0.372	0.258	-0.353	-0.265	-0.574	**1**	-0.373	0.006
Cost of sick leave (€)	0.135	0.360	0.312	-0.167	0.854	0.409	0.506	0.389	0.336	0.300	-0.373	**1**	0.121
Cost productivity loss (€)	-0.021	**0.866**	**0.877**	**-0.780**	-0.342	**0.839**	0.512	**0.842**	**0.870**	-0.087	0.006	0.121	**1**
